# Green Catalysis: The Role of Medicinal Plants as Food Waste Decomposition Enhancers/Accelerators

**DOI:** 10.3390/life15040552

**Published:** 2025-03-28

**Authors:** Liziwe L. Mugivhisa, Madira C. Manganyi

**Affiliations:** Department of Biological and Environmental Science, Sefako Makgatho Health Sciences University, P.O. Box 139, Ga-Rankuwa, Pretoria 0204, South Africa; liziwe.mugivhisa@smu.ac.za

**Keywords:** composting, decomposition, food waste, green catalysts, organic waste management secondary metabolites, sustainable practices

## Abstract

The escalating global issue of food waste, valued at billions of USD annually and significantly impacting sustainability across social, economic, and environmental dimensions, necessitates innovative solutions to enhance waste management processes. Conventional decomposition techniques frequently encounter challenges related to inefficiencies and extended processing durations. This investigation examines the potential contributions of medicinal plants as green catalysts in the decomposition of food waste, utilizing their bioactive compounds to mitigate these obstacles. Medicinal plants facilitate the decomposition process through various mechanisms as follows: they secrete enzymes and metabolites that aid in the disintegration of organic matter, enhancing microbial activity and soil pH and structure. Furthermore, they foster nitrogen cycling and generate growth regulators that further optimize the efficiency of decomposition. The symbiotic associations between medicinal plants and microorganisms, including mycorrhizal fungi and rhizobacteria, are also instrumental in enhancing nutrient cycling and improving rates of decomposition. The utilization of medicinal plants in food waste management not only accelerates the decomposition process but also underpins sustainable practices by converting waste into valuable compost, thereby enriching soil health and lessening dependence on chemical fertilizers. This methodology is congruent with the 2030 Agenda for Sustainable Development and presents a plausible trajectory toward a circular economy and improved environmental sustainability.

## 1. Introduction

In an era characterized by high mass production and consumerism, it is not surprising that we are experiencing the highest levels of food waste in history. Food waste is currently valued at billions of USD per annum, accounting for 14% of losses between harvest and the retail market globally [1]. Additionally, 17% of food is wasted at the retail and consumer levels [2]. Food waste affects sustainability across social, economic, and environmental dimensions, including the waste of raw materials, cleaning supplies, energy consumption, and water footprint [3]. It leads to the excessive consumption of freshwater and fossil fuels, contributing to global climate change through methane and CO_2_ emissions [4]. Accumulating evidence indicates that food wastage is a complex, multi-faceted issue involving various parameters, necessitating multiple strategies [5,6]. Nevertheless, a thorough examination of the possible solutions to this matter necessitates the consideration of the 2030 Agenda for Sustainable Development. A key objective of the Sustainable Development Goals (SDG) is the reduction in food wastage by half at the retail, consumption, and production levels through the advocacy and implementation of the 3R initiatives (reduce, reuse, recycle) [7].

**Figure 1:**
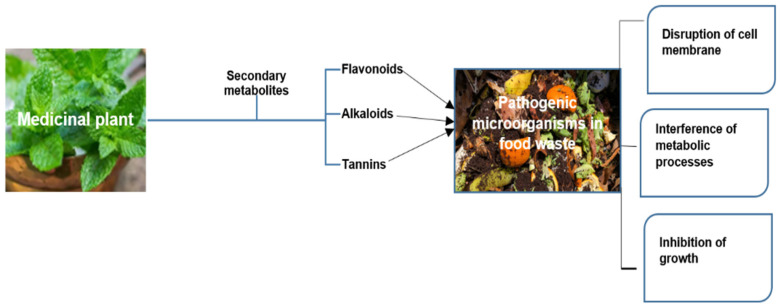
Mechanism of the antimicrobial properties of medicinal plants to selectively kill pathogenic microorganisms (constructed by L.L. Mugivhisa).

The decomposition of food waste is a crucial process for sustainable and eco-friendly waste management. Once organic waste undergoes appropriate composting, it degrades into compost that is abundant in nutrients, suitable for enhancing soil quality and plant development [8]. This procedure not only redirects waste away from landfills, thereby diminishing the release of methane—a potent greenhouse gas—but also reintroduces essential nutrients to the soil, completing the nutrient cycle [9,10]. Through the adoption of these sustainable approaches, we can diminish the impact on the environment, decrease dependence on chemical fertilizers, and contribute to the establishment of a more circular economy [11]. On the other hand, the turnover time for decomposition presents a significant shortcoming. To overcome these shortcomings and meet sustainable goals, green catalysis using medicinal plants provides a breakthrough alternative. A growing body of evidence has shown that medicinal plants serve as promising green catalysis in the decomposition of food waste [10,11,12,13]. These plants generate a wide variety of bioactive substances that have the ability to accelerate the decomposition of organic materials and improve the effectiveness of the process. By using medicinal plants as catalysts, the process becomes more environmentally friendly, accelerating decomposition and lowering the need for chemical additives [14]. Furthermore, the antimicrobial properties of medicinal plants can help kill pathogenic microorganisms during decomposition [15], as shown in Figure 1.

**Figure 2:**
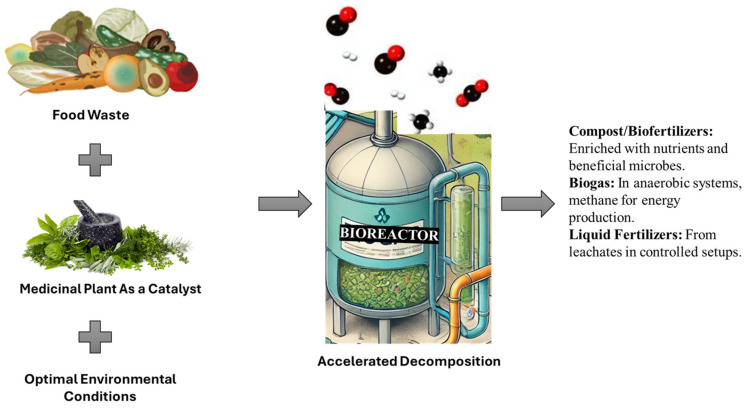
Graphical abstract on utilizing medicinal plants and food waste for sustainable decomposition and bioresource recovery (constructed by M.C. Manganyi).

The green innovative approach not only addresses the issue of prolonged decomposition times but also contributes to a circular economy by transforming food waste into valuable compost, thereby enriching soil health and reducing landfill use. The resource recovery of valuable food waste that is the target of circular economy includes compost/biofertilizers with nutrients and microbes which are beneficial for the breakdown of organic matter in the soil, enhancing the fertility of the soil (Figure 2). Another valuable resource is the biogas emanating from the anaerobic decomposition process and the methane which is crucial for the production of energy. Additionally, there is also the release of liquid fertilizers emanating from the leachates. Liquid fertilizer, which is also referred to as compost tea, is a good source of organic matter, nutrients, crucial microorganisms and their metabolic products.

This review aims to critically evaluate the potential of medicinal plants as green catalysts in food waste decomposition, emphasizing their bioactive compounds and mechanisms of action in accelerating organic matter breakdown. By integrating their enzymatic activity, microbial interactions, and soil-enhancing properties, medicinal plants present a sustainable alternative to conventional methods of organic waste degradation. Understanding these mechanisms provides a foundation for optimizing food waste management strategies aligned with global sustainability goals (Figure 2).

## 2. Food Waste Management Challenges

Food is a crucial product that is important for our survival. Contrary to food being an essential requirement for humans and 821 million people not having access to adequate food required for a healthy and active life globally, food waste is a major threatening global issue in achieving a sustainable future [16,17,18]. Therefore, food waste and food loss should be afforded serious consideration and tackled to reduce their incidence and the effect on humans, as well as the total balance of the environment and the ecosystem [19]. The present state of escalating quantities of food waste has resulted in a swing of economic, social, and sustainability issues such as climate change and environmental consequences that need an immediate and imperative solution and attention [19,20]. In some countries, food waste may account for 50% of the overall produced solid waste [9,21]. Food waste from food outlets such as restaurants, grocery stores, and households has become an increasing social problem in recent years, leading to roughly 1.3 billion tons of food waste being discarded into landfills per year, as reported by the Food and Agriculture Organization [22,23,24,25]. Global food waste constitutes a threat to food security through its impact on food availability, climate change, land, water, and the economy, as it increases food prices and tightens markets and biodiversity [18]. As a result, vulnerable communities, in particular women and children, are affected the most, leading to global inequalities [18].

The growing population, hectic life schedules, and chaotic living styles have resulted in drastic changes in the styles of food consumption, which have in turn resulted in large quantities of food being left over and wasted [26]. As a result of the increasing generation of waste due to the increase in the population, fast urbanization, thriving economy, and rising standards of living, there has been an urgent need to address the increasing environmental issue of global food waste [27]. Food waste was expected to increase by 30% in 2020, because people bought more than they needed due to the booming economy and healthier lifestyles [28]. An estimated food waste amounting to one-third of food produced results along the food supply chain [24,29]. The United Nations International Children’s Emergency Fund (UNICEF) stated that 1 billion people suffered from chronic hunger and 21,000 died as a result of problems related to hunger in 2011 [16], while in 2018, over 820 million people suffered from hunger [25]. Over 3.1 billion people and about 783 million people could not afford a healthy diet in 2021 and faced hunger around the world, respectively [30], while 1 in every 9 people went to bed starving every night, with the majority of those people in Asia and Africa [18].

The number of hungry people has increased as a result of global food wastage and this mars global food security in that there is a reduction in food availability and resources for food production for future generations [18]. As a result of this growing problem of food waste, it has been stated in the SDG 12.3 Global Food Loss and Waste that by 2030, the food waste per capita globally would have been halved at the consumer and retail levels, with food losses along the production, supply chain, and postharvest levels [17]. About 1.8 million tons of food waste was generated every year by British households. It is estimated that 45% of the total food supply is wasted in South Africa [17], and about 10.3 million tons of edible food was wasted in South Africa according to a five-year average which was taken from 2014 to 2018 [31]. Food waste, which is a by-product resulting from almost every household daily due to human use, represents a crucial challenge in waste management [28]. It accounts for more than 60% of discarded waste [32]. The fate of these large quantities of wasted food is landfill, where it rots and contributes to the emissions of methane, which is a more potent greenhouse gas than carbon dioxide, contributing to climate change [30,33]. Without the efficient management of food waste, dumping food waste results in the pollution of the environment, including water and air pollution, which negatively affect the functioning of ecosystems and human health [28]. The challenge with food waste is that it cannot be recycled or sorted since it contains large amounts of water, amounting to about 74.5% [32]. As a result, food waste could be used for biological treatment and decomposition at a later stage [33].

Food waste refers to biodegradable food that is not used, wasted, not eaten, inedible, expired, or left to spoil and ends up being discarded in the food supply chain during the stages of preparation, consumption, and distribution [34]. According to Lahiri et al. [19], food waste can be categorized into different groups depending on its nature. Food waste can be unintentional or intentional, unavoidable, partially avoidable, or avoidable. Unintentional food waste results from the loss or decline in the quantity or quality of food due to impairments or limitations during storage, processing, harvesting, or distribution, leading to food loss, whereas intentional food waste results from any food being disposed of or discarded from the food supply chain before use [35]. As a result, food waste is regarded as a postharvest phenomenon. As a result of the growing problem of food waste, a variety of systems to utilize food waste in a sustainable manner have been implemented. Even though there has been an increasing number of studies on the appraisal of food loss waste in previous years, there are still major challenges with regards to the reliability of the data and insufficiencies in the chronological and geographical reporting of food supply chains.

## 3. Current Methods of Food Waste Decomposition

**Figure 3:**
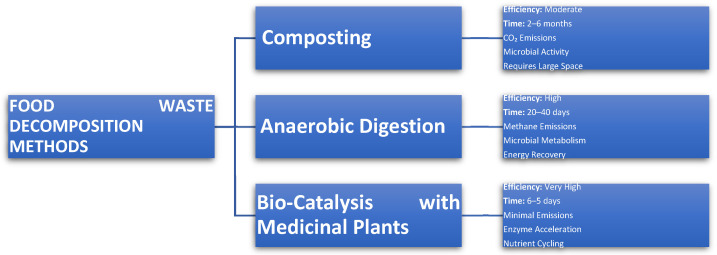
Comparative analysis of food waste decomposition technologies.

Figure 3 shows a comparative analysis of food waste decomposition technologies. With food waste making up 45% and 55% of the total municipal solid waste in European and developing countries, respectively, the ultimate end point of food waste has either been incineration or landfills until a couple of years ago [9]. Even though this still happens in most countries, more sustainable waste management methods and legislation have been considered in other countries. The conventional methods of incineration and landfill dumping, which are cost-effective and simple, have been commonly used as management practices to address and handle pilling food waste [28]. However, these methods are unsustainable and contribute to pollution in the environment through the emission of greenhouse gasses and groundwater pollution through leachate contamination and the emission of flue gas [23]. Due to the harmful and enormous effects of food waste, the treatment technologies of food waste need to be cheap, portable, affordable, economical, socially acceptable, environmentally friendly, energy efficient, stable, and with easy maintenance [32].

### 3.1. Anaerobic Digestion

In landfills, the anaerobic digestion of organic food wastes releases biogas consisting of carbon dioxide, methane, and small amounts of hydrogen sulfide, nitrogen, and oxygen [33]. Most landfills have reached their full carrying capacity, and the composting of food waste at landfills by anaerobic digestion takes long [28]. The anaerobic digestion method is simple and has a low capital cost, even though it is associated with bacteria inhibition due to exposure to toxins in the food waste [33]. Other than the prolonged periods of the microbial reaction, which takes about 20–40 days for the anaerobic digestion of organic food waste rich in nitrogen-rich proteins, there is also the production of high levels of free ammonia, which is harmful to the action of methanogenic bacteria, resulting in the toxic effects of the anaerobic digestion of food waste [33]. Anaerobic digestion (AD) is a multi-step microbial process that transforms organic waste into biogas, primarily methane, through intricate chemical pathways. The process relies on four main microbial groups—hydrolyzers, acidogens, acetogens, and methanogens—working in concert to degrade organic matter. Syntrophic relationships, including direct interspecies electron transfer (DIET), play a vital role in enhancing microbial cooperation and methane production. Key metabolic pathways such as hydrolysis, acidogenesis, acetogenesis, and methanogenesis facilitate the breakdown of carbohydrates and proteins, optimizing waste treatment. Ethanol also acts as an electron precursor, promoting interspecies electron transfer and enhancing acetate formation, which methanogens utilize to generate methane. The efficiency of AD is influenced by environmental factors such as pH, temperature, and substrate composition, which shape microbial community dynamics. While optimizing microbial interactions and pathways is crucial for improving AD performance, the stability of microbial communities and environmental constraints remain significant challenges to achieving consistent biogas production [33]. Even though the enriched digestates emanating from this anaerobic process can be used as soil fertilizers or soil conditioners, anaerobic digestion is also accompanied by gasses released into the atmosphere, contributing to climate change and environmental pollution [36]. Anaerobic digestion (AD) is recognized as a low-cost technology for waste management and renewable energy production. It is economically viable due to its ability to treat a wide range of organic materials, producing biogas and other value-added products. This process is particularly beneficial in rural and developing areas where resources are limited and waste management is a challenge. The low-cost nature of AD is attributed to several factors, which are discussed below.

Economic viability: AD systems are capable of efficiently treating both solid and liquid waste, reducing the need for more expensive waste management solutions [37]. The process generates biogas, which can be used for electricity, heat, or as a vehicle fuel, providing a cost-effective energy source. The use of low-cost materials, such as plastic bag digesters, makes AD accessible for small-scale applications in rural communities [38].

Technological aspects: AD technology is adaptable, with various reactor designs available to suit different environmental conditions and waste types. The process is environmentally friendly, reducing greenhouse gas emissions and contributing to a circular economy by converting waste into energy.

In developing countries, small-scale AD systems have been optimized for local conditions, demonstrating their feasibility and cost-effectiveness [38]. Self-designed low-cost reactors have been successfully used for biogas production from organic waste, such as sheep manure, highlighting the potential for low-budget implementations [39].

### 3.2. Composting

Composting has been used as a way of disposing food waste and recycling organic matter to enhance soil fertility and structure [40]. The technique of composting has been considered to be a sustainable and affordable way of managing the food waste challenge [28]. It is a cheap, eco-friendly, and economically sustainable alternative used to produce organic fertilizer from food waste and has been given extensive global attention, dealing with reuse and waste disposal problems simultaneously [41,42]. Composting is widely regarded as an environmentally friendly method for managing organic waste, offering numerous ecological benefits. It transforms organic materials into nutrient-rich compost, which enhances soil quality, reduces landfill waste, and mitigates greenhouse gas emissions. However, challenges such as malodorous gas emissions during the composting process must be addressed to fully realize its potential. The following sections elaborate on the key aspects of composting’s environmental friendliness.

❖ Waste reduction: Composting organic waste from landfills, reducing methane emissions associated with anaerobic decomposition [43].
❖ Soil enhancement: The resulting compost improves soil structure, fertility, and moisture retention, promoting sustainable agriculture [44].
❖ Greenhouse gas mitigation: Composting can significantly lower greenhouse gas emissions compared to traditional waste disposal methods [43].

Challenges and Considerations

❖ Odorous emissions: The release of volatile organic compounds (VOCs) during composting, particularly ammonia and sulfur compounds, poses health risks and environmental concerns [45].
❖ Management techniques: Strategies such as optimal aeration and the addition of bulking agents can mitigate these emissions, enhancing the overall sustainability of composting [45]. While composting is generally seen as eco-friendly, it is essential to address its challenges to maximize its environmental benefits. Balancing effective waste management with odor control remains a critical area for ongoing research and improvement.

The principle of food waste processing and degradation entails biological decomposition which happens over a prolonged period of more than 30 days. This involves methods which are expeditious, inexpensive, eco-friendly, socially tolerable, and easy to maintain [32]. Composting duration varies significantly based on the methods and materials used. Traditional composting typically takes between two to six months, depending on the environmental conditions and the composition of the organic materials [46]. However, innovative techniques can drastically reduce this time frame.

Traditional Composting Duration

❖ Time frame: Generally, composting takes from 2 to 6 months.

Factors influencing duration:

❖ Ambient temperature.
❖ Moisture content (ideally 30–35%).
❖ Carbon-to-nitrogen (C:N) ratio (should be less than 20) [46].

Research shows that using activators like EM4 can reduce composting time to as little as 10 days while achieving a favorable C:N ratio of 11 [47]. Segmented aerobic composting: This method can shorten composting to just 72 h by effectively controlling the temperature stages [40]. Hydro-thermal treatment: This advanced method combines hydro-thermal treatment with post-decomposition, taking only 10–15 days [47]. Conversely, some methods, such as traditional rotting, can extend composting from one to two years, highlighting the variability in composting practices and their efficiency. According to the research findings, the duration of “2 to 6 months” is the general consensus.

In 2006, 4 million tons of the organic portion of the Municipal Solid Waste (MSW) in the European Union was treated to produce organic fertilizer through composting by 124 compost facilities, while in 2005, Spain, the Netherlands, and France used 33%, 24%, and 14% of their total waste to produce compost, respectively [48].

Composting is a biological process which takes place under aerobic conditions in the presence of oxygen [48]. It is an environmentally friendly and vigorous technology used for treating organic waste into valuable nutrients and organics, which are end products with high value [23]. Different constituents in organic waste are broken down by the biochemical process of composting into moderately stable humus-like substances that can be utilized as organic fertilizers [48]. In order for the quality of the compost to be high, the compost production requires the management and control of the moisture content, particle size, aeration, pH, carbon-to-nitrogen ratio, and porosity [9]. Composting involves a process of stabilizing organic matter under aerobic conditions, using microorganisms where organic matter originating from plants and animals is degraded into more stable materials that are more hygienic and have shorter molecular chains and a high humus content, contributing to the fertility of agricultural lands and soil organic matter recycling [49]. Additionally, the compost improves soil structure and tilt conditions, enhances the ability of the soil to hold nutrients and water, supports living organisms in the soil and the biological control of particular soil pests, and returns organic matter to soils while keeping the organic materials out of waterways and landfills [49].

#### 3.2.1. Natural Fermentation

The current methods of composting include the natural fermentation method, which uses the aerobic process that accelerates the biodegradable food waste transformation into a rich useful organic compost that can be crucial for crop farming [50]. Through an aerobic process, organic compounds are oxidized to nitrite, nitrate, and carbon dioxide in the presence of oxygen [48]. The natural fermentation method enhances microbial growth, with microbial activities speeding up the process of decomposition [51]. The source of energy is carbon, while organic compounds are degraded by thermophilic bacteria and recycled to give off macronutrients, such as potassium (K), nitrogen (N), phosphorus (P), carbon (C), and hydrogen (H), in the compost, offering suitable growing conditions for plants [52].

The natural fermentation composting process involves the use of a mixture of cultures of advantageous microorganisms such as *Bacillus*, *Pseudomonas*, and *Azotobacter*, which have a significant potential for controlling and inhibiting phytopathogenic organisms, hence creating a soil environment that is favorable for the protection and growth of plants [53]. This correlates with factors which include the generation of metabolic heat, moisture content, ventilation, and temperature [27]. For the fermentation process to be effective, the intensity of the activity of the microorganisms must be maintained within a range that is desirable and is reflected by the temperature range [54]. With the appropriate microbial activity control, microbial growth rate, and food waste moisture, the food waste fermentation process can be optimized due to the speedy production of enzymes by microorganisms and the enhanced degradation of food waste [27]. In turn, the high degradation rate contributes to the production of more energy, which increases temperature, resulting in the enhancement of the composting process [55].

#### 3.2.2. Open Windrow Composting

Open windrow composting entails the mixing and stacking of raw materials into windows which are long and narrow, or piles which become agitated and remixed regularly and continuously to expose all material to temperature, light, and air, through a blower, with the process taking between 12 and 20 weeks to complete [56]. The composting materials are put on the uppermost perforated pipe or platform while air is blown upwards or sucked downwards, with sucked aeration assisting in the removal of odors. The formed pile requires turning or agitation, resulting in the active stage being completed in 3 to 5 weeks [57].

#### 3.2.3. In-Vessel Composting

In in-vessel composting, compost is produced in channels or drums making use of a controlled aeration system at high rates to produce the optimal conditions for microbes, contributing to the enhancement of organic matter decomposition [18]. The required aeration is accomplished through the continuous aeration of the compost by using various mechanical turning techniques [58]. The advantage of in-vessel composting is that it provides conditions which are optimum for the microbes, enhancing the decomposition process and resulting in a mature compost within a short period of time, with better control compared to other methods due to the high efficiency of the process [18,59,60].

#### 3.2.4. Vermicomposting

Vermicomposting is another effective method of composting which entails the use of worms that break down the complex organic compounds into smaller and simpler molecules. The red worms change the decaying organic matter into worm castings with high nutrient concentrations which are crucial for the growth of plants [61]. Composting uses earthworms, in particular, African night crawlers and red wigglers, which are great decomposers. Earthworms act as important facilitators, increasing the accessibility of the surface area to microorganisms to increase the action of the enzymes and to change the physical attributes of the organic waste [62]. Vermicompost contains a variety of enzymes, such as chitinase, cellulase, amylase, and lipase, which assist with splitting the organic matter to release the nutrients to the roots of plants [52]. The secretion of chemicals from the worm’s digestive tracts helps break down organic matter, so the product has a higher nutrient saturation than its original form. This type of composting is particularly used when food waste is made up of table and kitchen scraps. However, thermophilic conditions cannot be achieved with this type of composting, as worms are unable to survive at high temperatures [48].

### 3.3. Bio-Drying

Another method of processing food waste which has been found to be effective through the acceleration of the compost maturity process entails using bio-drying technology together with a mixture of bulking agent in the form of stable and mature compost and microorganisms [32]. Bio-drying, also referred to as biological drying, is the most interesting way of managing food waste decomposition [63]. Bio-drying uses the mechanical process of air circulation and biological drying, whereby a bio-drying reactor is used to process the chopped food waste with high water content, resulting in the food waste compost that has been dried. The microbial decomposition of organic matter generates metabolic heat, which is essential for evaporating moisture during bio-drying. This activity is heavily influenced by oxygen availability and aeration control [64]. The key chemical pathways involved in bio-drying include the oxidation of nitrogen-containing compounds, resulting in the accumulation of nitrate nitrogen [65], and the degradation of lipids and hydrolysable substances, which facilitate the release of bound water. Proteins and polysaccharides are subsequently degraded as secondary substrates [64]. Maintaining high temperatures (up to 70 °C) and controlled aeration is essential for optimizing microbial activity and moisture evaporation [65]. Additionally, energy-efficient ventilation strategies, such as intermittent ventilation, enhance temperature accumulation and moisture removal, thereby improving the efficiency and cost-effectiveness of the bio-drying process [64,65]. The advantage of bio-drying is that it enhances stabilization and reduces the water content, weight, volume, and odor of food waste. This, in turn, improves handling, transporting, and minimizing the disposal of food waste from households, while producing low emissions of carbon dioxide, which mitigate the effects of climate change. In this type of accelerated composting, the raw material which is used together with the mature compost is made up of 60% leaf waste and 40% manure [32].

Composting and anaerobic digestion are influenced by key environmental factors such as temperature, microbial diversity, moisture retention, and nutrient cycling. Medicinal plants enhance these processes by introducing bioactive compounds that optimize microbial activity, regulate pH balance, and accelerate decomposition. These compounds, primarily secondary metabolites, exhibit various beneficial properties that contribute to ecological and health-related processes. Medicinal plants contain bioactive compounds such as phenolic acids and flavonoids, which have demonstrated significant antibacterial activity against pathogens like *Escherichia coli* and *Salmonella enteritidis* [66]. The antimicrobial properties of plant extracts can help maintain a balanced pH in various environments, promoting optimal conditions for microbial activity [67]. By regulating pH, these compounds can enhance nutrient solubility, further supporting microbial growth and activity [68]. Bioactive compounds facilitate the breakdown of organic matter, accelerating decomposition processes essential for nutrient cycling in ecosystems [69]. The interaction between medicinal plant compounds and microbial communities can lead to increased efficiency in decomposition, benefiting soil health and fertility [68]. For instance, bioactive compounds in neem (*Azadirachta indica*) and tulsi (*Ocimum sanctum*) promote microbial proliferation, which leads to the faster breakdown of organic matter. Furthermore, composting enriched with medicinal plant extracts has demonstrated increased nitrogen availability and reduced methane emissions compared to traditional anaerobic digestion, thus reducing the environmental footprint of food waste management.

Table 1 presents a comparative analysis of various food waste decomposition methods, highlighting their respective advantages, disadvantages, and environmental impacts to provide a comprehensive overview of their effectiveness and sustainability. From all the composting methods, natural fermentation is the best method which is more affordable since it uses an aerobic process in the presence of oxygen, which is an easily accessible and freely available commodity. In addition, microbial growth and activity in the natural fermentation process speed up the decomposition process and the energy for steering the process comes from the carbon in organic compounds from food waste. Macronutrients, which are essential in the compost and play a role in promoting plant growth, are also released during the natural fermentation process of composting.

## 4. Medicinal Plants as Green Catalysts for the Decomposition of Food Waste

The advancement of biotechnological research has spurred progressive innovation in bio-catalysis, also known as green catalysts. Their application within various sectors aims to accelerate chemical reactions without being consumed in the process, thereby enhancing efficiency and reducing energy consumption. Notably, these catalysts originate from natural sources. Their most compelling aspect lies in their characteristics of low toxicity, biodegradability, and sustainability, aligning with the current trend of eco-friendly green living [73]. In this context, medicinal plants are a suitable green catalyst candidate for the decomposition of food waste due to their ability to fulfill the criteria through various mechanisms. For instance, they produce secondary metabolites that can be utilized in metal complex catalysis, as well as cytochrome P450 enzymes (CYPs). Additionally, cytochrome P450 enzymes frequently act as essential catalysts in metabolite biosynthesis.

A study conducted by Shul’ts et al. [74] demonstrated that enzymes such as eudesmane-type methylidenelactones, diterpenes, alkaloids, furanolabdanoids, triterpenoids, and coumarins act as metal complex catalysts in plants. Meanwhile, cytochrome P450 (CYP450) enzymes oxidize various organic compounds during the biosynthesis of hormones and the detoxification processes. Overall, an ordered and synchronized catalytic cycle begins with the CYP450 catalyzing the reaction [75]. Another aspect of medicinal plants as green catalysts is the presence of various compounds such as flavonoids and polyphenols, which possess antioxidant properties. These compounds play a crucial role in catalyzing oxidation-reduction reactions during the breakdown of organic substances. Fighting oxidative stress and boosting plants’ antioxidant capabilities require the action of enzymes such as superoxide dismutase (SOD), peroxidase (POD), polyphenol oxidase (PPO), and ascorbate peroxidase (APX) [76].

Medicinal plants employ new solid acid catalysts that come from natural sources to facilitate hydrolysis and other acid-catalyzed reactions. One example of a natural mild catalyst with solid acid properties has been found to be effective is horsetail and horsetail ash, exhibiting high catalytic activities in a variety of events [77]. Furthermore, it has been shown that ionic liquids made from quaternary ammonium salts are useful as acidic catalysts for carboxylic acid esterification reactions and organic ester hydrolysis [78,79]. To ensure high activity at moderate temperatures and long-term use, it has also been suggested to use solid oxide supports containing radicals of phosphoric acid or sulfate as long-lasting catalysts in the hydrolysis of plant-based materials [80]. The various ways that acid catalysts derived from medicinal plants are used to promote hydrolysis and other acid-catalyzed reactions are demonstrated by the combined findings of these investigations.

## 5. Mechanisms of Action: How Medicinal Plants Accelerate Decomposition

A growing body of evidence suggests that medicinal plants play a significant role in accelerating decomposition through complex biological and physical mechanisms [81,82,83]. These mechanisms include the rhizosphere priming effect, where root exudates from medicinal plants stimulate microbial activity, leading to enhanced organic matter decomposition. Additionally, medicinal plants secrete secondary metabolites that modulate soil enzyme activity, further promoting organic matter degradation [84]. Furthermore, the production of phytotoxic decomposition byproducts by these plants can disrupt soil microbial communities and reduce inter-plant competition, creating an environment favorable for rapid decomposition [85]. Taken together, these processes emphasize the pivotal role of medicinal plants in regulating nutrient availability and ecosystem functioning.

### 5.1. Enzymes in Decomposition

Enzymes in medicinal plants play a crucial role as catalysts in the decomposition of plant materials, facilitating various biochemical processes. These enzymes, particularly those from fungi and plants, are essential for breaking down complex biopolymers, contributing to both natural decay and potential biotechnological applications. Enzymes such as cellulases, proteases, and ligninases are instrumental in degrading cellulose, proteins, and lignin, respectively [86]. The action of these enzymes transforms complex organic molecules into simpler, more bioavailable forms, thereby accelerating the decomposition process. For instance, cellulases break down cellulose into glucose, which can then be utilized by soil microorganisms, enhancing their activity and promoting further decomposition [87]. This enzymatic activity is crucial for nutrient cycling in ecosystems, as it not only aids in the breakdown of organic matter but also improves the availability of essential nutrients for other plants and organisms in the soil [88].

Research has shown that the production of these enzymes is not limited to the plants themselves but is also significantly influenced by the associated microorganisms, such as endophytic fungi. These fungi, which reside within the tissues of medicinal plants, can produce extracellular enzymes that complement the plant’s own enzymatic activity [89]. For example, studies have demonstrated that endophytic fungi isolated from various medicinal plants can secrete enzymes like amylase, cellulase, and protease, which further enhance the decomposition of organic materials [90,91]. The synergistic action of plant and fungal enzymes produces a robust system for organic matter degradation, illustrating the complex interactions within the rhizosphere that contribute to ecosystem health. Furthermore, certain endophytic fungi have been found to exhibit higher cellulolytic activity, which can significantly enhance the breakdown of lignocellulosic biomass [92]. Endophytic fungi, such as *Aspergillus niger* and *Cladosporium* sp., have demonstrated significant capabilities in producing various extracellular enzymes, including amylase, protease, and cellulase [93,94].

Cellulases and hemicellulases are hydrolytic enzymes that play a crucial role in breaking down the complex carbohydrates found in plant cell walls, such as cellulose and hemicellulose. These enzymes work synergistically to depolymerize the long chains of glucose and other sugars that make up these structural polysaccharides [95,96]. By cleaving glycosidic bonds, cellulases and hemicellulases facilitate the access of other microorganisms to the organic matter, making it more readily available for decomposition [97].

Cellulases can be divided into three main classes as follows: endoglucanases, exoglucanases, and β-glucosidases. Endoglucanases randomly cleave internal bonds in the cellulose chain, creating new chain ends. Exoglucanases cleave off cellobiose units from the reducing and non-reducing ends of the cellulose chain. Finally, β-glucosidases hydrolyze cellobiose and short cellooligosaccharides into glucose monomers [98,99]. Hemicellulases, on the other hand, target the diverse array of hemicellulose structures, including xylan, mannan, and glucan backbones, with various side chains [100,101]. Proteases are enzymes that catalyze the hydrolysis of peptide bonds in proteins, breaking them down into smaller peptides and amino acids. This process makes the nitrogen-containing compounds more readily available for microbial assimilation and incorporation into their biomass [102,103]. Proteases can be classified based on their catalytic mechanism (e.g., serine proteases and cysteine proteases) or their site of action (e.g., endoproteases and exoproteases) [104,105].

Lipases are enzymes that catalyze the hydrolysis of ester bonds in lipids, breaking them down into fatty acids and glycerol. This process makes the carbon and energy stored in lipids more accessible for microbial metabolism [106,107]. Lipases can act on a wide range of lipid substrates, including triglycerides, phospholipids, and glycolipids, and are produced by various microorganisms and plants [106,107,108,109]. Oxidative enzymes, such as peroxidases and laccases, can degrade certain pesticides and pollutants by catalyzing their oxidation. This process can reduce the toxicity of these compounds and facilitate their further breakdown by other microorganisms [110,111]. Peroxidases use hydrogen peroxide as an electron acceptor, while laccases use molecular oxygen [112,113].

Oxidative enzymes, particularly phenol oxidases and peroxidases, contribute to the formation of humus, a stable organic matter component essential for soil health. These enzymes catalyze the oxidation of phenolic compounds, leading to the formation of quinones, which can undergo further polymerization and condensation reactions to produce humic substances [114,115]. Humus improves the soil structure, water-holding capacity, and nutrient availability, supporting plant growth and overall ecosystem functioning [116,117]. These processes are essential for nutrient cycling and the maintenance of soil health in terrestrial ecosystems.

### 5.2. Secondary Metabolite in Decomposition

Secondary metabolites produced by medicinal plants play a pivotal role in accelerating decomposition processes in ecosystems through various mechanisms that enhance microbial activity and organic matter breakdown. These metabolites, which include alkaloids, phenolic compounds, and terpenoids, significantly influence soil microbial communities and nutrient cycling. Secondary metabolites can alter the composition and activity of microbial communities in the soil. The secondary compound hypothesis suggests that secondary plant metabolites (SPMEs) released through root exudation or litter decomposition can stimulate the growth of specific microbial populations that are efficient in degrading complex organic materials. For example, Chomel et al. [118] have demonstrated that specific phenolic compounds can promote the growth of microbial taxa that specialize in breaking down recalcitrant litter, which is often rich in secondary metabolites.

Secondary metabolites can modulate the activity of the soil enzymes involved in the degradation of organic matter. For instance, phenolic compounds have been shown to enhance the activity of lignin-degrading enzymes, facilitating the breakdown of tough plant materials [119]. This enzymatic enhancement is crucial for effective decomposition, as it allows microorganisms to access and utilize the carbon and nutrients stored in complex organic compounds [120]. The presence of secondary metabolites can promote the release of nutrients from decomposing organic matter. By enhancing microbial activity and enzyme production, these compounds contribute to the accelerated mineralization of nutrients, making them more available for plant uptake [121]. This nutrient release is vital for maintaining soil fertility and supporting plant growth, further contributing to cycling organic matter within the ecosystem.

Secondary metabolites also have broader ecological implications. They can influence trophic interactions within the soil food web, affecting not only microbial communities but also soil fauna such as earthworms and nematodes [122]. These interactions can lead to cascading effects that enhance decomposition rates and improve soil health over time. Research indicates that litter derived from the same habitat as the soil community (home-field advantage) decomposes faster than litter from foreign habitats. This phenomenon is thought to result from the local adaptations of the soil microbial communities to decompose the specific types of litter they encounter most frequently, particularly those rich in secondary metabolites [123]. This adaptation allows for more efficient decomposition processes, as local microbial communities are better equipped to handle the biochemical properties of familiar litter types. Many medicinal plants produce antimicrobial compounds like alkaloids, flavonoids, and terpenes. While these compounds inhibit the growth of pathogenic microorganisms, they can also create an environment conducive to the growth of beneficial decomposer microorganisms, such as fungi and bacteria, which enhance decomposition.

### 5.3. Stimulation of Microbial Activity

Medicinal plants play a crucial role in enhancing decomposition by stimulating microbial activity through the release of bioactive compounds. These compounds, including terpenoids, flavonoids, and other secondary metabolites, influence microbial dynamics, promoting the growth of beneficial microbes while suppressing harmful ones. This selective stimulation of microbial communities is essential for efficient nutrient cycling and the breakdown of organic matter in ecosystems. Bioactive compounds in medicinal plants act through multiple mechanisms to support microbial activity. For instance, compounds from *Terminalia chebula* possess antimicrobial properties that can selectively inhibit pathogens while promoting beneficial microbial populations, which are key to maintaining a balanced soil microbiome and enhancing decomposition [124]. In addition, these compounds can stimulate the production of critical enzymes like cellulases and ligninases, which accelerate the breakdown of organic material [125]. Furthermore, natural products often act on multiple targets within microbial cells, increasing metabolic activity and thereby boosting decomposition rates [126].

The mechanisms through which medicinal plant compounds act are diverse and impactful. They can alter microbial membrane permeability, enhancing nutrient uptake and proliferation—both crucial for efficient decomposition [127]. Moreover, some bioactive compounds interfere with bacterial quorum sensing, disrupting pathogen communication and allowing decomposer microbes to thrive [128]. However, this microbial stimulation is not without risk, as certain antimicrobial properties may unintentionally suppress beneficial species, leading to imbalances [129]. Based on our current understanding, medicinal plants not only offer therapeutic benefits but also play a significant ecological role in stimulating microbial activity for enhanced decomposition. Their bioactive compounds contribute to a dual benefit in both health and environmental sustainability, highlighting their potential for ecological applications.

### 5.4. Modification of Soil pH and Structure

Medicinal plants significantly influence soil decomposition processes through a variety of mechanisms, particularly by modifying soil pH and altering microbial community dynamics. Understanding these interactions is essential for improving soil health and optimizing nutrient cycling in ecosystems. Soil pH plays a critical role in determining the rates of litter decomposition, with the pH of the plant’s leaf and litter often exerting a greater influence on decomposition than the overall soil chemistry [130]. In acidic soils, plant species traits, such as leaf pH, can profoundly impact microbial activity and community composition, leading to varying rates of organic matter breakdown. The acidic environment alters microbial functioning by favoring certain species and enzymes, ultimately shaping the decomposition process [130].

Soil pH also directly influences the composition and dynamics of microbial communities. Bacterial populations tend to thrive in higher pH soils, whereas fungi dominate in more acidic conditions [131]. This microbial shift has a pronounced effect on decomposition, as fungi are generally more efficient decomposers of complex organic materials, such as lignin and cellulose. Therefore, pH-driven changes in microbial dominance can accelerate or slow down decomposition, depending on the microbial composition [131]. Medicinal plants contribute to enhanced soil nutrient levels, particularly increasing concentrations of nitrates and phosphorus compared to conventional crops. This nutrient enrichment fosters microbial activity, further promoting decomposition processes [132]. Moreover, medicinal plant soils often exhibit a lower pH, which can enhance the activity of specific microbial groups, resulting in faster organic matter breakdown [133].

Medicinal plants significantly enhance decomposition processes by modifying the soil structure through several key mechanisms. They contribute to soil aggregation by releasing root exudates that bind soil particles, improving soil porosity, and facilitating microbial activity. The organic matter from medicinal plants, such as leaf litter and root biomass, enriches soil humus, increasing soil fertility and stability, which supports more efficient decomposition [133]. Additionally, their extensive root systems alleviate soil compaction, enhancing soil aeration and microbial access to organic materials. Furthermore, the structural complexity provided by medicinal plants creates diverse microbial habitats, fostering a rich community of decomposers [133]. These combined effects of improved soil structure and microbial dynamics lead to accelerated decomposition and more effective nutrient cycling, demonstrating the critical role of medicinal plants in promoting ecosystem health.

### 5.5. Promotion of Nitrogen Cycling

**Figure 4:**
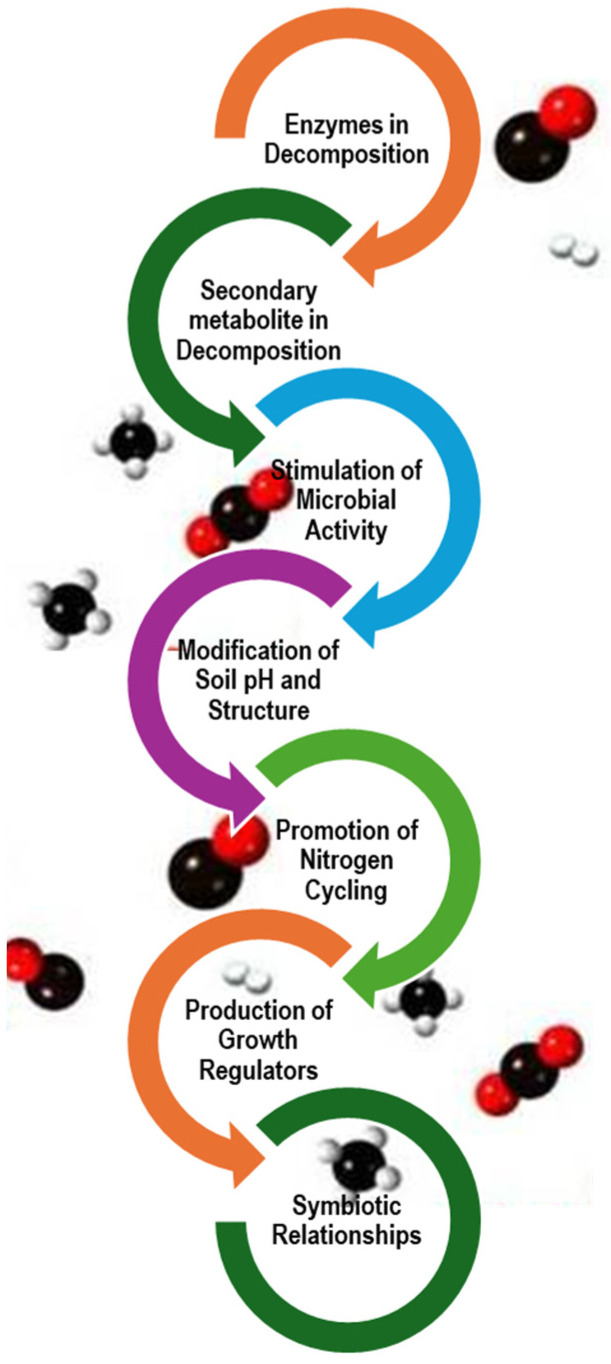
Mechanisms of action responsible for the acceleration of decomposition (constructed by M.C. Manganyi).

With regard to nitrogen cycling, medicinal plants play a crucial role in accelerating decomposition through mechanisms such as enhanced nutrient uptake, microbial interactions, and improved litter quality. These processes collectively contribute to more efficient nutrient turnover, promoting ecosystem health and productivity. Nitrogen uptake by medicinal plants is a key factor in the decomposition process. Elevated nitrogen deposition enhances plant nutrient absorption, leading to higher nitrogen content in plant litter, which in turn accelerates decomposition rates [134] [Figure 4]. The quality of the litter, particularly its nitrogen content, significantly influences decomposition dynamics, with higher nitrogen levels promoting faster breakdown and more rapid nutrient release into the soil [135]. This accelerated decomposition enriches the soil, fostering improved nutrient cycling.

In addition to direct nitrogen uptake, medicinal plants modify the structure and function of soil microbial communities, particularly by enhancing the abundance of nitrifying and denitrifying microorganisms that are essential for nitrogen cycling [136]. The carbon compounds supplied by plant roots stimulate microbial activity, creating a nitrogen loop where microbes facilitate both nitrogen mineralization and immobilization. This interaction directly influences overall nitrogen dynamics within the ecosystem, ensuring that nitrogen is efficiently cycled and made available for plant and microbial use [125]. However, while medicinal plants contribute positively to nitrogen cycling and decomposition, the potential for excessive nitrogen inputs poses a risk to ecosystem health. Elevated nitrogen levels can lead to negative environmental consequences, such as nutrient leaching and water pollution [137]. Therefore, managing the balance of nitrogen inputs and cycling is critical for maintaining sustainable ecosystem function and preventing adverse environmental impacts.

### 5.6. Production of Growth Regulators

Growth regulators enhance decomposition processes in medicinal plants by modulating various physiological and biochemical pathways. These regulators, such as auxins and cytokinins, are critical in accelerating plant growth and the production of secondary metabolites, which play essential roles in ecological interactions and decomposition. For example, indole-3-acetic acid (IAA) and benzylaminopurine (BAP) stimulate cell division and elongation, leading to increased biomass. This enhancement in plant growth can improve litter quality and accelerate decomposition rates [138]. Additionally, the application of growth regulators boosts the synthesis of secondary metabolites, which not only enhance plant resilience but also contribute to nutrient cycling within ecosystems [139]. The increased plant growth and secondary metabolite production foster the stimulation of soil microbial communities, further accelerating decomposition processes [140]. However, while growth regulators significantly impact decomposition, their excessive use can lead to ecological imbalances. Therefore, sustainable application practices are essential in medicinal plant cultivation to maintain ecological stability and promote effective nutrient cycling.

### 5.7. Symbiotic Relationships

Medicinal plants significantly accelerate decomposition through their symbiotic relationships with various microorganisms, which enhance nutrient cycling and improve the efficiency of organic matter breakdown. Fungal endophytes, such as Epichloë, interact with grasses to accelerate litter decomposition by altering the quality and quantity of plant tissues, thus enhancing decomposition rates and modifying nitrogen dynamics in the decomposed litter [141]. Similarly, fungus-growing termites, in association with Termitomyces fungi, employ a combination of carbohydrate-active enzymes and oxidative reactions, including Fenton chemistry, to effectively decompose lignin-rich plant biomass. This tripartite symbiosis involving termites, fungi, and gut microbiomes is crucial for efficient biomass conversion and nutrient cycling [142]. Additionally, plant growth-promoting rhizobacteria (PGPR) form beneficial relationships with medicinal plants, promoting growth and resilience against abiotic stresses. These rhizobacteria indirectly influence decomposition by enhancing plant health, which affects the quantity and quality of the plant litter available for decomposition [143]. Collectively, these symbiotic interactions significantly enhance the decomposition processes of medicinal plants, optimizing nutrient cycling and contributing to ecosystem sustainability. By improving litter quality and quantity and by employing diverse enzymatic and oxidative mechanisms, these relationships facilitate the more efficient breakdown of plant biomass. Understanding these interactions not only highlights the role of medicinal plants in accelerating decomposition but also offers valuable insights into managing ecosystem health and promoting sustainable practices.

## 6. Case Studies: Successful Applications of Medicinal Plants in Food Waste Management

Although composting is a highly valued process in managing waste, due to its vigor and potential to produce valuable products with a potential for fertilizing the soil for plant growth, the challenge with composting is that it requires a long period of time for mature compost to be produced [9]. Since composting food waste is crucial, efforts have been made to improve its efficiency to alleviate the associated environmental degradation and reduce its operating costs [39]. It is crucial that stable and mature compost should be produced in a short period of time, using additives and amendments such as chemicals in the form of hormones, bulking agents, biochar from waste biomass, and mineral element-degrading microbes [43]. Although composting is a natural process that is developing fast, the incorporation of inoculating substances improves the rate of degradation of the organic matter so that there is a substantial decrease in the operation time for composting, and there should be an improvement in the quality of the compost [9]. Wiharyanto et al. [32] have shown that an introduction of local microorganisms and the bio-drying technology in Indonesia, contributed to the acceleration of the maturation process of the food waste compost, with the maturation phase being completed within three days. Even though up to 20–25% of solid waste can be decomposed with the aid of chemicals, there has been a need to degrade larger quantities of solid waste with the assistance of herbal medicines, which are affordable and make waste decomposition more financially effective [26]. Successful applications of medicinal plants in food waste management include the Chinese medicinal herbal residues comprising fibrous plant materials, which contain high levels of carbon that can be utilized as a bulking material for composting [140]. The Chinese medicine herbal residues contain phytochemicals which might increase the quality of the compost. This can be achieved through enhanced antipathogenic properties due to abiotic and biotic mechanisms with the bioactive substances emanating from flavonoid and alkaloid groups in Chinese medicinal herbal residues. As a result, there is the possibility of influencing the composting process through the control of the microbe populations [23]. The use of chemicals for the degradation of food waste, which makes up the largest portion of solid organic waste, includes herbal drugs that have also been shown to be efficient in degrading food waste within shorter periods of time in India [26].

According to Ayare et al. [26], in India, organic food waste was degraded with the aid of medicinal plants, including aloe vera, asafetida, tulsi, and neem. The powder of asafetida is a popular home remedy used for treating bloating in India, and together with buttermilk, it has health benefits. The leaves, seeds, seeds oils, gum, root, and stem barks of neem have been used in Ayurvedic remedies and medicines from ancient times for a variety of medicinal purposes [135]. Tulsi has been used medicinally for the maintenance of celibacy and lessening the ill effects of radiotherapy in cancer treatment, whereas the benefits of aloe vera are only restricted to cosmetics [136]. Even though these Ayurvedic medicinal plants, which are available in households and in nature, were recommended for food waste degradation since they resulted in a considerable reduction in the volume and weight loss of the treated organic food waste, asafetida was ineffective in the degradation of food waste, as it took over 20 days to decompose 1 kg of food waste and also required more water for additional degradation and decomposition. Aloe vera was also shown to be ineffective, as it was associated with the growth of fungus and also needed more water for the decomposition process. Nevertheless, both neem and tulsi were found to be more effective and economical, with a degradation time of 6 days and 90% degradation of food waste compost [26]. Table 2 summarizes the decomposition properties of medicinal plants, including their bioactive compounds, mechanisms, environmental benefits, decomposition time, and related challenges. It also highlights case studies of their effectiveness in sustainable practices.

According to Zhou et al. [23], during the extraction and decoction processes of Chinese medicinal herbs, less than 50% of the bioactive compounds are extracted, leaving behind a significant quantity of active ingredients. The increase in the herbal residue ratio has been shown to result in a positive effect of extending the thermophilic phase by 9 days [23]. The addition of Chinese medicinal herbal residues resulted in an enhanced decomposition of organic material. The incorporation of a microbial inoculum present in Chinese medicine herbal residue composting showed an increase in the efficacy of the degradation of cellulose [23]. The integration of Chinese medicine herbal residues showed a positive impact on food waste bulking, as a result of the enhanced degradation of the organic content, resulting in Chinese medicine herbal residues being recommended for the composting of food waste to produce a mature compost enriched with nutrients [23]. When Chinese Medicinal Herb Residues (CMHRs) were used as co-compost with food waste, ref. [137] observed that the utilization of CMHRs resulted in an anti-pathogenic compost due to the improved antipathogenic properties of the plants, protecting against infection, and the degradation of the organic matter and the acceleration of the humification process were accelerated by CMHRs. The microbe population, together with their mycoparasitic and antagonistic properties, were influenced by the presence of CMHR-derived active ingredients, resulting in the inhibitory effect of the FW-CMHR compost against pathogens [137]. This showed that composts based on CMHR can be a possible biological anti-pathogenic agent, concurrently providing the nutrients required by the plants, hence adding value to the product of the compost [137].

## 7. Benefits and Challenges of Using Medicinal Plants in Food Waste Decomposition

One of the biggest challenges in modern-day living is sustainability. Hence, there is a need for processes, techniques, and materials that elicit less negative environmental impacts, while promoting efficiency, profitability, and sustainability [143]. The employment of medicinal plants and their products or extracts in enhancing food waste decomposition assists in reducing or eradicating the use of toxic and volatile substances. The medicinal plants’ extracts provide efficient and environmentally friendly substances and techniques, which eliminate or reduce the release of harmful toxic chemical substances into the environment during the decomposition of food waste. Medicinal plants provide an economic advantage in enhancing food waste decomposition in that they do not need to be processed, as they can be used in their natural form. As a result, the expenses associated with processing and production are decreased, contributing to higher profits.

Potential barriers to the adoption of medicinal plants for enhancing the decomposition process of food waste are over-harvesting and the exploitation of medicinal plants, with some of these medicinal plants being indigenous and endemic to South Africa alone. The exploitation of these medicinal plants can threaten biodiversity and ecological sustainability. The consumption of the medicinal plants can also be subjected to social resistance from environmental activists or activists protecting heritage and the indigenous knowledge of medicinal plants. Since medicinal plants are used for drug development, their use might encounter resistance due to the economic advantages associated with harnessing beneficial crude extracts for drug development. In addition, with the advent of COVID-19 and other pandemics, there has been a lot of research focusing on the antibacterial and anti-microbial properties of medicinal plants and their products to combat the spread of infections and diseases.

The challenge of the toxicity of medicinal plants on agricultural land and cultivated crops due to phytochemicals can be mitigated through the use of other soil amendments like bioachar and clay particles, in addition to the compost. Clay particles have been shown to have adsorbent characteristics for pollutants or toxins in the soil [144].

## 8. What Is the Way Forward?

With the rapidly increasing global population, there is a need for enhanced food production that employs sustainable soil amendments to enhance plant growth through, for example, composting the escalating food waste that has a negative impact on the environment. However, the composting process requires prolonged periods of time for the compost to mature. Green catalysis in the form of products from medicinal plants enhancers can assist in speeding up the composting process so that food production can increase. Nevertheless, parallel to that, more research should focus on processes or methods which use sustainable, innovative, and unconventional sources of energy for the decomposition of food waste to combat the challenge of escalating food waste that continues to pile up in landfills and produces toxic environmental impacts, such as the emission of greenhouse gases and leaching leading to the contamination and pollution of groundwater. Efforts should also go into process management and engineering techniques that achieve energy efficiency during the decomposition of waste.

## 9. Conclusions and Perspective

The increasing prevalence of food waste poses substantial obstacles for sustainability, affecting the environmental, social, and economic spheres. Conventional decomposition methods, although advantageous, frequently fail to adequately address the extended turnover times and inefficiencies that characterize the existing processes. The utilization of medicinal plants presents an innovative solution through the mechanism of green catalysis, significantly enhancing the efficiency of food waste decomposition. By harnessing their various bioactive compounds, medicinal plants can facilitate accelerated decomposition, improve nutrient cycling, and result in a more sustainable framework of waste management. The contribution of medicinal plants in this regard is complex and varied. They play a pivotal role in the decomposition process through several mechanisms, such as the synthesis of enzymes and secondary metabolites that aid in the disintegration of organic matter. Moreover, they enhance microbial activity, alter soil pH and structure, promote nitrogen cycling, and generate growth regulators that collectively improve decomposition efficiency. The symbiotic associations established by medicinal plants further optimize these processes, thereby fostering a resilient and effective decomposition system.

This green catalytic approach not only addresses the pressing concern of extended decomposition durations but also aligns with the objectives outlined in the 2030 Agenda for Sustainable Development. By incorporating medicinal plants into food waste management frameworks, it is possible to convert waste into valuable compost, enhance soil health, reduce dependence on chemical fertilizers, and reduce landfill utilization. The endorsement of medicinal plants as catalysts in the decomposition of food waste signifies a noteworthy advancement towards a circular economy and a more sustainable future.

## Figures and Tables

**Table 1:** Comparative analysis of food waste decomposition methods: advantages, disadvantages, and environmental impacts.

Decomposition Method	Advantages	Disadvantages	Environmental Impact	Refs.
Anaerobic Digestion	Low capital cost, simple process, produces biogas (methane and CO_2_) as an energy source. Can be used as soil fertilizers.	Long decomposition period (20–40 days). Inhibition of bacteria by toxins. High ammonia levels harmful to methanogenic bacteria. Initial costs are usually quite high.	Contributes to greenhouse gas emissions (methane). Potential environmental pollution through leachate.	[70,71]
Composting	Eco-friendly. Inexpensive. Produces valuable organic fertilizers. Enhances soil structure and fertility.	Slow process (2 to 6 months). Requires management of moisture, aeration, pH, etc. Needs space for composting facilities. Odorous emissions (Tran et al., 2024 [45]).	Reduces landfill waste. Helps with soil regeneration. May release greenhouse gases (CO_2_).	[72,73,74]
Natural Fermentation	Accelerates decomposition. Enhances microbial growth. Produces nutrient-rich compost for farming.	Requires control of microbial activity. Requires temperature, moisture, and ventilation management.	Reduces food waste. May release heat and CO_2_ into the atmosphere.	[53,55]
Open Windrow Composting	Simple to implement. Low cost. Allows for aeration and temperature control during decomposition.	Slow process (12–20 weeks). Requires large area. Needs frequent turning and aeration.	Reduces organic waste. Potential odor emissions. Releases CO_2_ and other gases during decomposition.	[56]
In-Vessel Composting	Faster decomposition. Better control over temperature and aeration. Produces mature compost quicker.	Higher initial setup cost. Requires more space. Mechanical systems needed for aeration.	Reduces food waste. Decreases landfill use. Releases CO_2_ and possible odors.	[60]
Vermicomposting	Fast decomposition. Produces nutrient-rich worm castings. Improves soil health. Eco-friendly.	Cannot handle high-temperature food waste. Not suitable for large-scale operations.	Reduces waste and produces high-nutrient organic fertilizer. Minimal environmental pollution.	[58]
Bio-Drying	Accelerates composting process. Reduces water content and volume. Low emissions of CO_2._ Improves handling and transport of waste.	Requires use of bulking agents like leaf waste and manure. May not be suitable for all types of food waste.	Reduces food waste volume. Lower CO_2_ emissions compared to traditional methods. May still emit some gases.	[63]

**Table 2:** Mechanisms of decomposition and environmental benefits of medicinal plants in food waste management.

Medicinal Plant	Key Bioactive Compounds	Decomposition Mechanism	Environmental Benefits	Decomposition Timeframe	Limitations/Challenges	Case Studies	Refs.
*Neem* (*Azadirachta indica*)	Alkaloids, flavonoids, terpenoids	Enhances microbial activity; antimicrobial effects reduce pathogens	Reduces chemical fertilizer use; lowers pathogen load	6 days	Overharvesting risk due to widespread usage	Proven effective with 90% degradation in 6 days.	[26,136]
*Tulsi* (*Ocimum sanctum*)	Essential oils, polyphenols	Promotes microbial enzymatic activity and nutrient cycling	Improves compost quality; enhances microbial diversity	6 days	Sensitive to water availability	Effective decomposition with enhanced nutrient content.	[26,138]
*Chinese Herbal Residues*	Flavonoids, alkaloids	Accelerates humification and inhibits pathogens	Prolongs thermophilic phase; reduces pathogen activity	9 days	Requires proper ratio mixing with food waste	Extends thermophilic phase and improves compost quality.	[23,139]
*Aloe vera*	Polysaccharides, anthraquinones	Aids moisture retention during decomposition; fosters fungal growth	Increases soil organic matter; retains moisture	20+ days	Slower degradation; fungus growth needs control	Slower but contributes to compost moisture retention.	[26,138]
*Horsetail* (*Equisetum* spp.)	Silica, flavonoids, alkaloids	Natural acid catalysis; promotes enzymatic hydrolysis	High catalytic activity for organic matter breakdown	Varies (depending on method)	Limited data on large-scale application	Effective hydrolysis catalyst in smaller systems.	[60]
*Asafetida* (*Ferula assa-foetida*)	Resins, essential oils, tannins	Stimulates microbial activity; antimicrobial effects	Reduces pathogen load; improves microbial activity	20+ days	Ineffective without additional water or bulking	Long degradation time despite microbial stimulation.	[26]
*Senna* (*Cassia* spp.)	Anthraquinones, tannins, flavonoids	Promotes breakdown of lignocellulosic biomass	Enhances lignin degradation; boosts soil fertility	15 days	Can inhibit non-target microbial communities	Effective for cellulose and lignin degradation.	[60,63]
*Tulip Tree* (*Liriodendron tulipifera*)	Terpenoids, polyphenols	Enhances microbial enzyme production	Accelerates organic matter breakdown in acidic soils	10–14 days	Adapted to specific soil pH ranges	Notable for accelerating decomposition in acidic environments.	[26]
*Vernonia amygdalina*	Saponins, tannins, flavonoids	Produces iron nanoparticles; enhances microbial oxidation	Catalyzes reactions for nutrient release	7–10 days	Requires controlled conditions for maximum effect	Promising results in small-scale decomposition trials.	[11,137]

## Data Availability

Not applicable.
